# Supplementary material for: Silicon/Hard Carbon Composites Synthesized from Phenolic Resin as Anode Materials for Lithium-Ion Batteries
Source: Nanomaterials (Basel). 2025 Mar 17;15(6):455. doi: 10.3390/nano15060455 (PMC11944807; doi:10.3390/nano15060455)
Supplement: Supplementary file 1 [file nanomaterials-15-00455-s001.zip › nanomaterials-3503865-supplementary.pdf]

Supporting Information

Silicon/hard carbon composites synthesized from phenolic resin as anode materials for lithium-ion batteries

Yu-Hsuan Li,[1] S. Kishore Babu,[1] Duncan H. Gregory,[2] Soorathep Kheawhom,[3,4] Jeng-Kuei Chang[5,6] and Wei-Ren Liu[1,7,*]

1 Department of Chemical Engineering, R&D Center for Membrane Technology, Chung Yuan Christian University, 200 Chung Pei Road, Chungli District, Taoyuan City, 32023, Taiwan, R.O.C.

2 WestCHEM, School of Chemistry, University of Glasgow, Glasgow G12 8QQ UK

3 Department of Chemical Engineering, Faculty of Engineering, Chulalongkorn University, Bangkok 10330, Thailand

4 Center of Excellence on Advanced Materials for Energy Storage, Chulalongkorn University, Bangkok 10330, Thailand

5 Department of Materials Science and Engineering, National Yang Ming Chiao Tung University, 1001 University Road, Hsinchu 30010, Taiwan, R.O.C.

6 Institute of Materials Science and Engineering, National Central University, 300 Jhong-Da Road, Taoyuan 32001, Taiwan

7 Hierarchical Green-Energy Materials (Hi-GEM) Research Center, National Cheng Kung University, 1 University Road, Tainan 70101, Taiwan, R.O.C.

**E-mail address: WRLiu1203@gmail.com*

Tel: +886 3-265-4140; fax: +886 3-265-4199

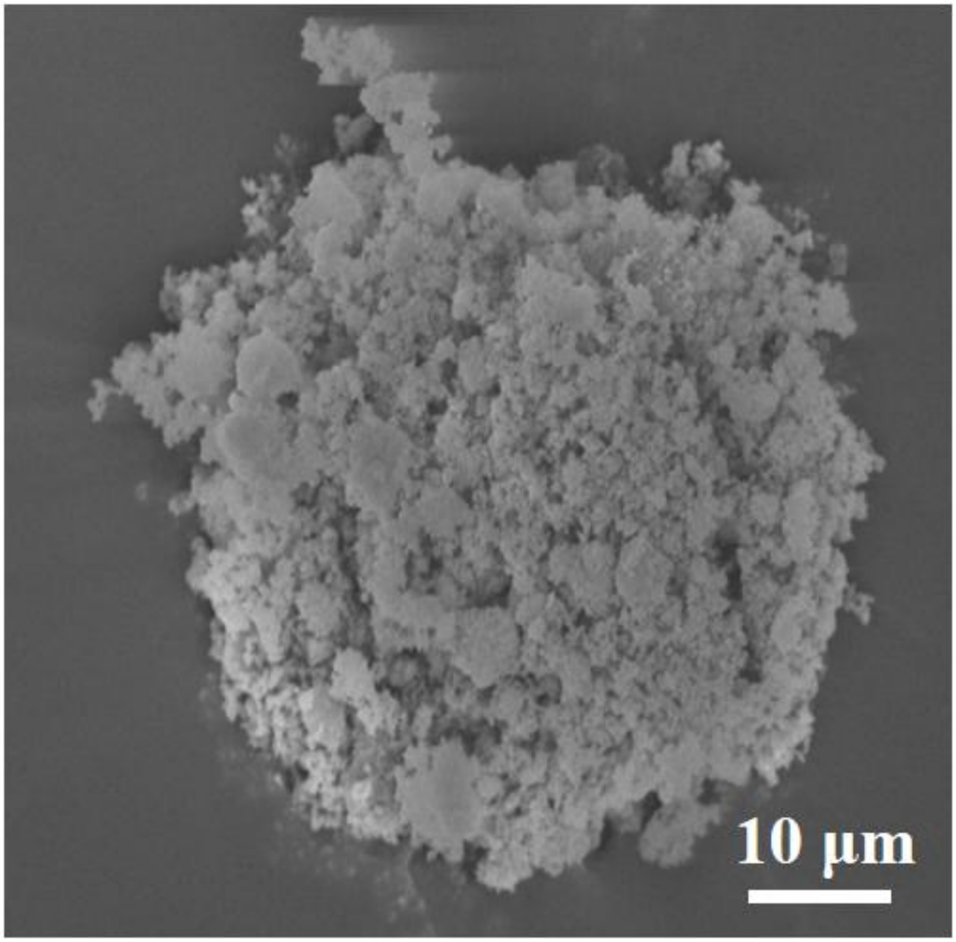

Figure. S1 SEM image of nano-silicon.

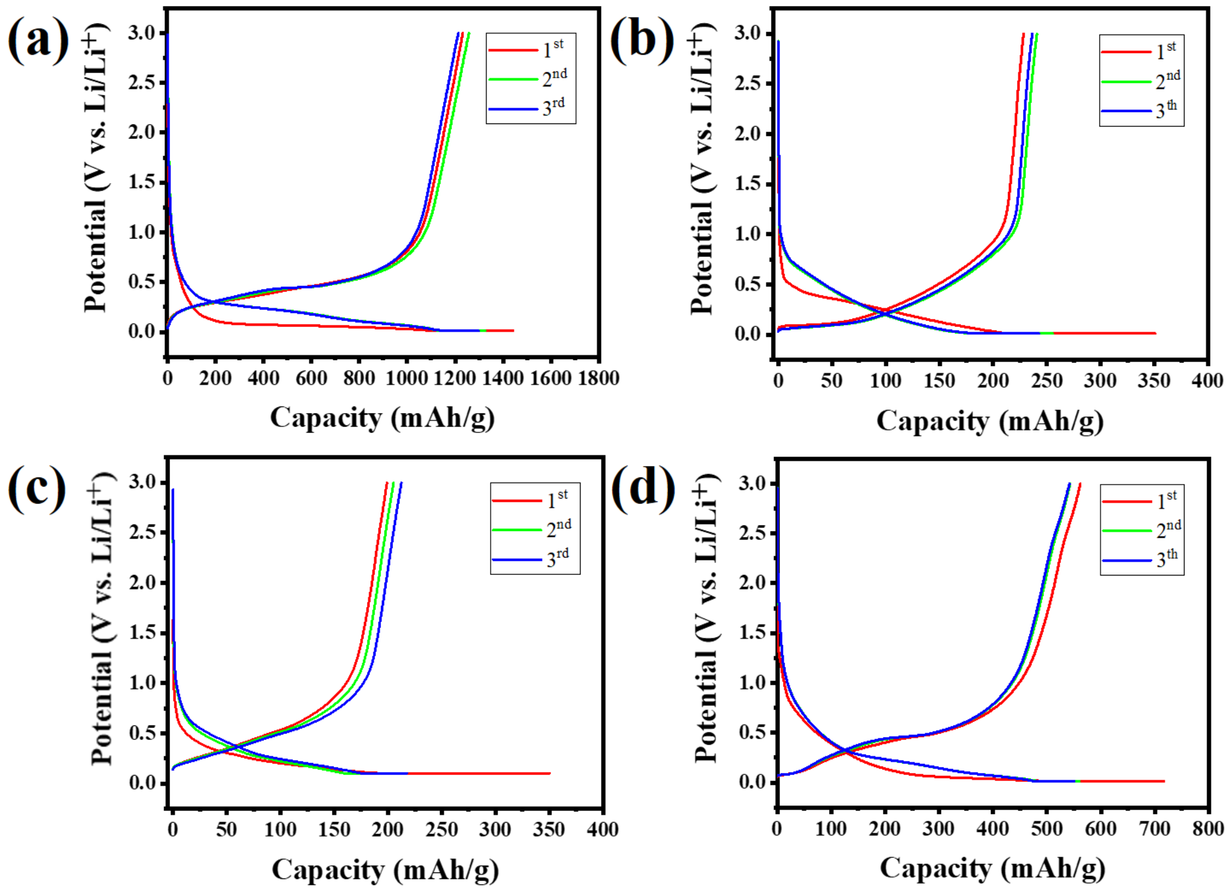

Figure S2 Charge/discharge curves of (a) Si, (b)HC, (c) HC@Si and (d) HC@Si-P at current density of 0.1 A/g for the initial three cycles.

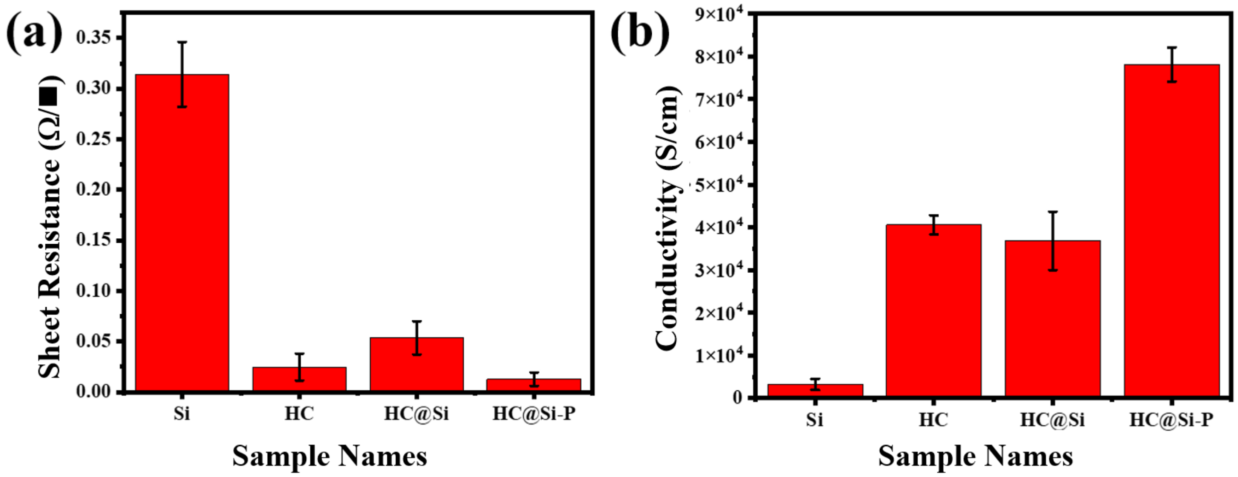

Figure S3 4-point probe tests of (a) Sheet resistances for Si, HC, HC@Si, and HC@Si-P electrode; (b) Conductivity for Si, HC, HC@Si, and HC@Si-P electrode.

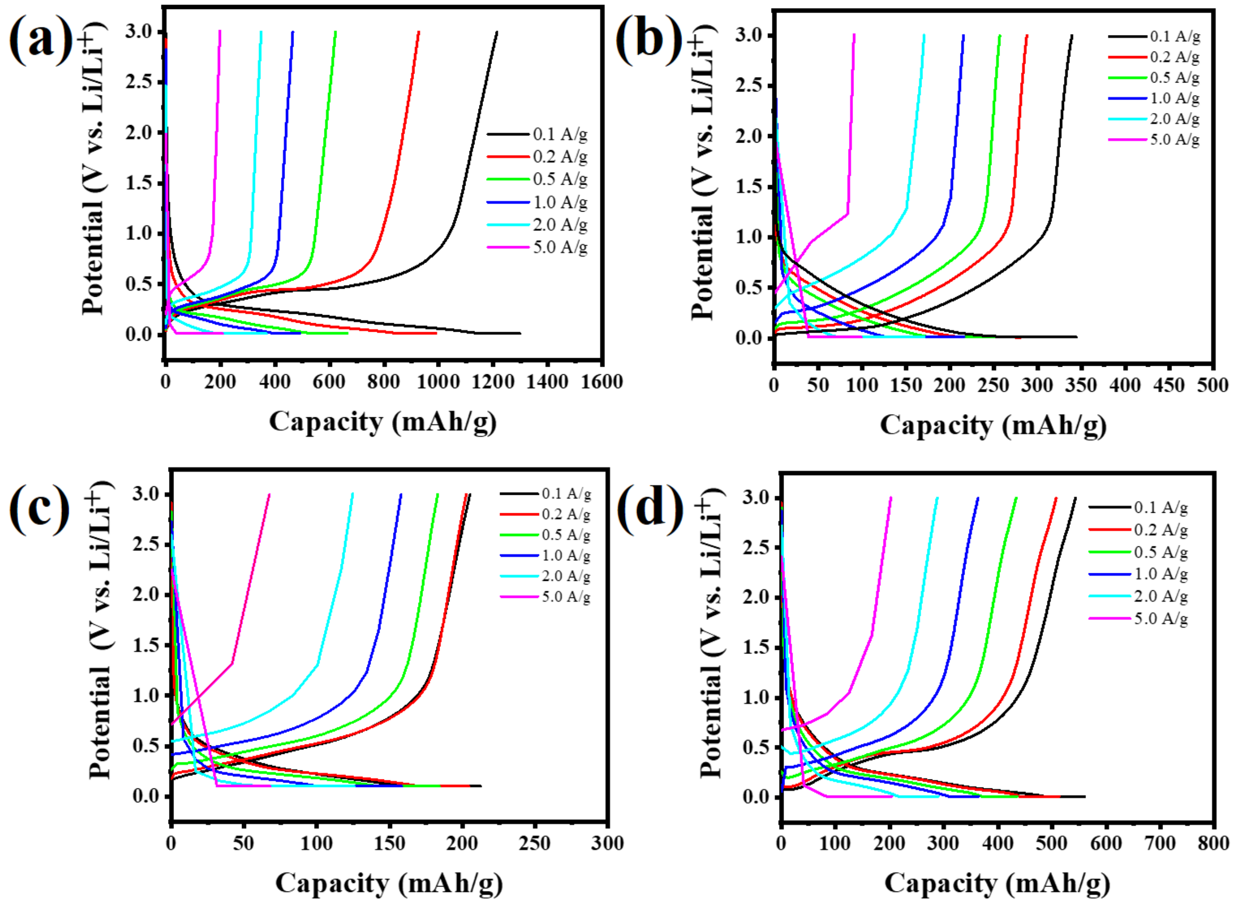

Figure S4 Charge/discharge curves of (a) Si, (b) HC, (c) HC@Si and (d) HC@Si-P at different current density.

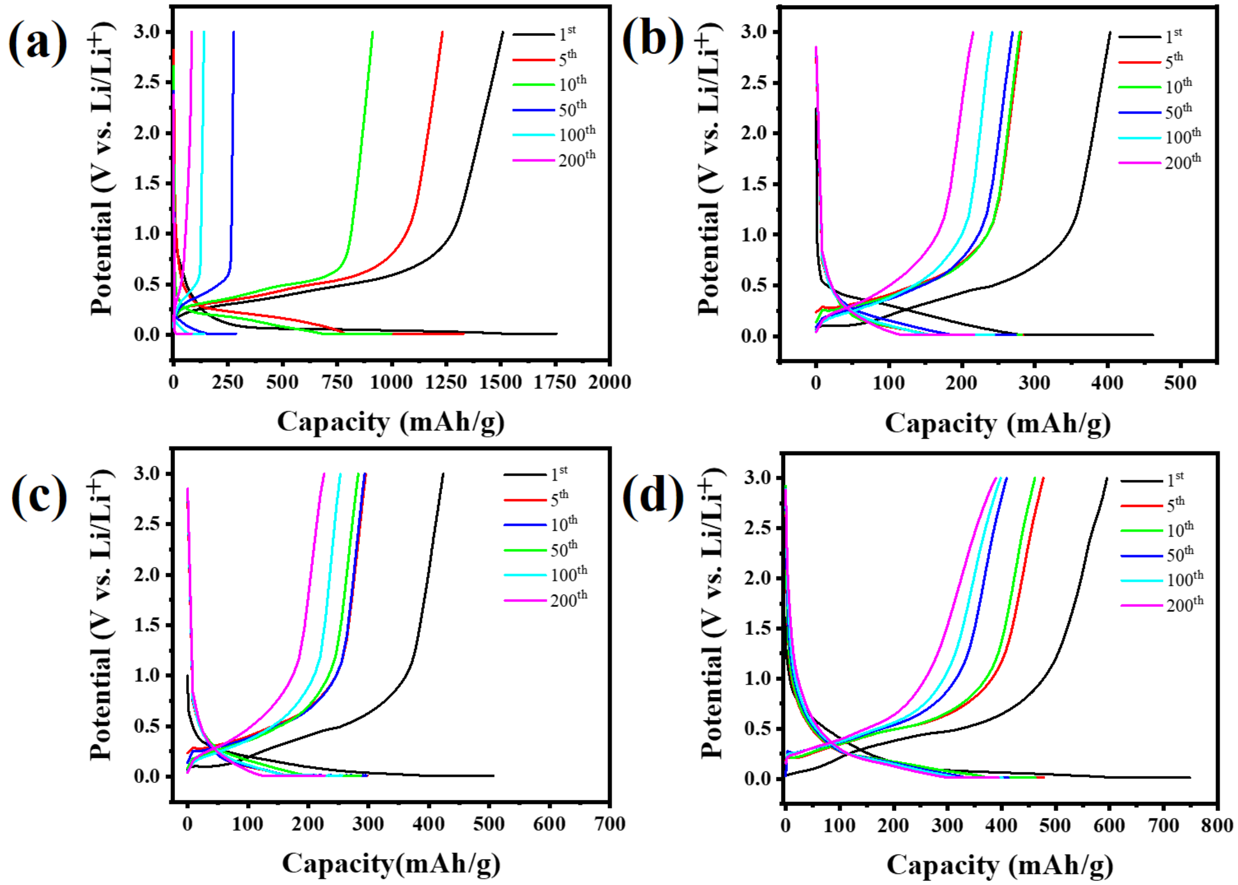

Figure S5 Charge/discharge profiles of (a) Si, (b) HC, (c) HC@Si and (d) HC@Si-P at different cycle under current density of 1 A/g. The first cycle of each sample was tested at 0.1 A/g.

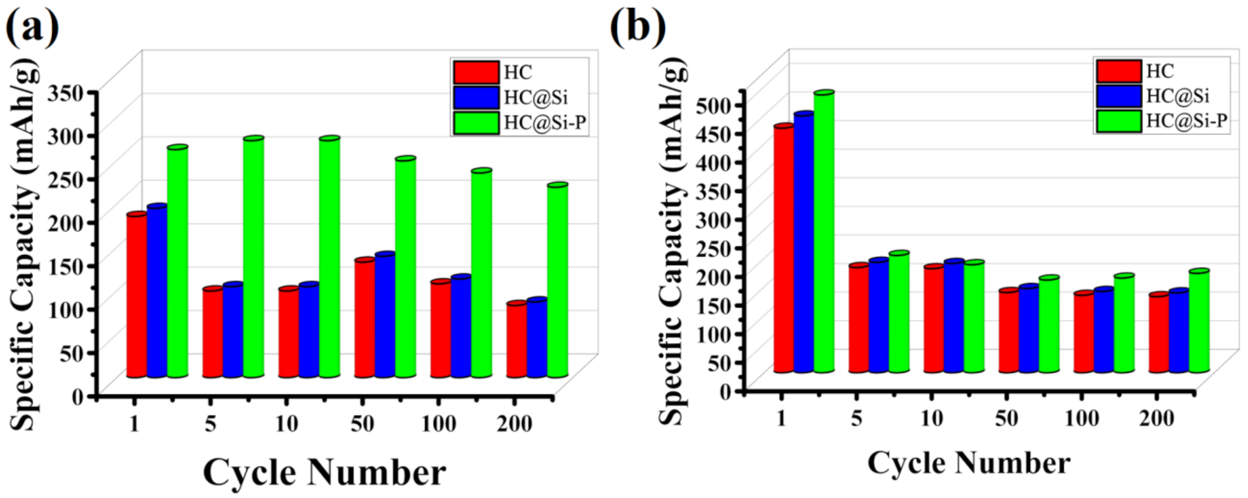

Figure S6 Lithium-ion storage of HC, HC@Si and HC@Si-P contributed from (a) slope capacity (> 0.1V) and (b) plateau capacity (< 0.1V) contribution analysis at different cycle.

Table S1. Electrochemical impedance, slope and diffusion coefficient of HC, HC@Si, and HC@Si-P.

**Samples**	**R_s (Ω)**	**R_{SEI} (Ω)**	**R_{CT} (Ω)**	**Slope**	**Diffusion coefficient (cm^2/s)**
**HC**	3.2	130.8	21.0	8.51	7.73×10^{-13}
**HC@Si**	6.7	118.3	45.1	6.58	1.38×10^{-12}
**HC@Si-P**	8.1	81.8	26.1	6.23	1.78×10^{-12}

Table S2. Comparison of electrochemical performance with reported Si/HC as anode materials for Lithium-ion batteries.

Samples	*ICE	After nth cycles	Capacity (mAh/g)	Current rate (A/g)	Ref.
**HC@Si-P**	**79%**	**200**	**391**	**1.0**	**This work**
Si/C	57%	50	678	0.1	[1]
Si@C	68%	500	868	0.1	[2]
Si@C-pitch	74%	100	629	0.5	[3]
Si@SiO_x@C	64%	50	770	0.2	[4]
SGC	79%	100	1526	0.25	[5]
Si@10C	72%	500	1006	0.5	[6]
Si@C@v@CNTs	76%	100	912	0.1	[7]
Si@Void@NC	74%	400	475.1	0.5	[8]
Si/C-AG	64%	200	445	0.5	[9]
Si@hNC	61%	100	735	0.2	[10]

*ICE: Initial Columbic Efficiency

References

1. Wang, M.; Fan, L. Silicon/carbon nanocomposite pyrolyzed from phenolic resin as anode materials for lithium-ion batteries. *J. Power Sources* **2013**, 244, 570-574.
2. Batool, S.; Idrees, M.; Kong, J.; Zhang, J.; Kong, S.; Dong, M.; Hou, H.; Fan, J.; Wei, H.; Guo, Z. Assessment of the electrochemical behaviour of silicon@carbon nanocomposite anode for lithium-ion batteries. *J. Alloys Compd* **2020**, 832, 154644.
3. Qi, Z.; Dai, L.; Wang, Z.; Xie, L.; Chen, J.; Cheng, J.; Song, G.; Li, X.; Sun, G.; Chen, C.Optimizing the carbon coating to eliminate electrochemical interface polarization in a high-performance silicon anode for use in a lithium-ion battery. *New Carbon Mater* **2022**, 37, 1, 245-258.
4. Hu, G.; Yu, R.; Liu, Z.; Yu, Q.; Zhang, Y.; Chen, Q.; Wu, J.; Zhou, L.; Mai, L. Surface oxidation layer-mediated conformal carbon coating on Si nanoparticles for enhanced lithium storage. *ACS Appl. Mater. Interfaces* **2021**, 13, 3991-3998.
5. Huang, Y.; Luoa, J.; Peng, J.; Shi, M.; Li, X.; Wang, X.; Chang. B.; Porous silicon–graphene–carbon composite as high-performance anode material for lithium-ion batteries. *J. Energy Storage* **2020**, 27, 101075.
6. Luo, W.; Wang, Y.; Chou, S.; Xu, Y.; Li, W.; Kong, B.; Dou, S.; Liu, H.; Yang, J. Critical thickness of phenolic resin-based carbon interfacial layer for improving long cycling stability of silicon nanoparticle anodes. *Nano Energy* **2016**, 27, 255–264.
7. Han, N.; Li, J.; Wang, X.; Zhang, C.; Liu, G.; Li, X.; Qu, J.; Peng, Z.; Zhu, X.; Zhang, L. Flexible carbon nanotubes confined yolk-shelled silicon-based anode with superior conductivity for lithium storage. *Nanomaterials* **2021**, 11, 699.
8. Wei, Y.; Huang, Y.; Zeng, Y.; Zhang, Y.; Cheng, W.; Wang, W.; Jia, D.; Tang, X.; Wang, L. Designed formation of yolk−shell-Like N-doped carbon-coated Si nanoparticles by a facile method for lithium-ion battery anodes. *ACS Appl. Energy Mater* **2022**, 5, 1471-1477.

9. Yang, W.; Ying, H.; Zhang, S.; Guo, R.; Wang, J.; Han, W. Electrochemical performance enhancement of porous Si lithium-ion battery anode by integrating with optimized carbonaceous materials. *Electrochim. Acta* **2020**, 337, 135687.

10. Yin, L.; Parka, M.; Jeon, Injun.; Hwang, J.; Kim, J.; Lee, H.; Park, M.; Jeong, S.; Cho, C. Silicon nanoparticle self-incorporated in hollow nitrogen-doped carbon microspheres for lithium-ion battery anodes. *Electrochim. Acta* **2021**, 368, 137630.
